# A Study on the Field Emission Characteristics of High-Quality Wrinkled Multilayer Graphene Cathodes

**DOI:** 10.3390/nano14070613

**Published:** 2024-03-30

**Authors:** Wenmei Lv, Lian Wang, Yiwei Lu, Dong Wang, Hui Wang, Yuxin Hao, Yuanpeng Zhang, Zeqi Sun, Yongliang Tang

**Affiliations:** School of Physical Science and Technology, Southwest Jiaotong University, Chengdu 610031, China; wenmei.lv@my.swjtu.edu.cn (W.L.); wl168@my.swjtu.edu.cn (L.W.); yiweilu@my.swjtu.edu.cn (Y.L.); dongwang1190@my.swjtu.edu.cn (D.W.); wanghui@swjtu.edu.cn (H.W.); haoyx@my.swjtu.edu.cn (Y.H.); zhangyuanpeng@my.swjtu.edu.cn (Y.Z.); 2017114679@my.swjtu.edu.cn (Z.S.)

**Keywords:** multilayer graphene, cathode, field emission, spin-coating method, screen-printed method

## Abstract

Field emission (FE) necessitates cathode materials with low work function and high thermal and electrical conductivity and stability. To meet these requirements, we developed FE cathodes based on high-quality wrinkled multilayer graphene (MLG) prepared using the bubble-assisted chemical vapor deposition (B-CVD) method and investigated their emission characteristics. The result showed that MLG cathodes prepared using the spin-coating method exhibited a high field emission current density (~7.9 mA/cm^2^), indicating the excellent intrinsic emission performance of the MLG. However, the weak adhesion between the MLG and the substrate led to the poor stability of the cathode. Screen printing was employed to prepare the cathode to improve stability, and the influence of a silver buffer layer was explored on the cathode’s performance. The results demonstrated that these cathodes exhibited better emission stability, and the silver buffer layer further enhanced the comprehensive field emission performance. The optimized cathode possesses low turn-on field strength (~1.5 V/μm), low threshold field strength (~2.65 V/μm), high current density (~10.5 mA/cm^2^), and good emission uniformity. Moreover, the cathode also exhibits excellent emission stability, with a current fluctuation of only 6.28% during a 4-h test at 1530 V.

## 1. Introduction

Field emission (FE) is a phenomenon whereby electrons are emitted by overcoming the surface potential barrier of a solid material through the quantum tunneling effect, induced by applying a sufficiently strong electric field [1]. FE is widely applied in various domains, including FE displays (FED) [2,3], gas sensors [4,5], microwave-generating devices [6,7], and X-rays [8]. Successful FE requires cathode materials with low work function and high thermal and electrical conductivity and stability. Graphene, a two-dimensional honeycomb-like carbon material, possesses high electrical conductivity [9], high thermal conductivity [10], and exhibits a stability against ion and electron irradiation similar to that of graphite. These exceptional properties have rapidly established graphene as a popular research area in FE materials.

One commonly used graphene material is reduced graphene oxide (RGO) [11,12], which usually has a high defect density, numerous oxygen functional groups on the surface, and multiple adsorption sites [13]. This leads to low thermal and electrical conductivity and a tendency to release gas during the emission process, significantly impacting its FE performance. For example, Eda et al. fabricated an RGO cathode with a current density of merely 10^−2^ μA/cm^2^ at a high electric field of 4 V/μm. The max current density (*J_max_*) obtained was 1 mA/cm^2^, with a field enhancement factor (*β*) of 1200 [11]. Qian et al. used RGO as the material and employed a screen-printed method to fabricate an FE cathode. The turn-on field corresponding to a current density of 1 μA/cm^2^ was 1.5 V/μm, and for 1 mA/cm^2^, the corresponding threshold field (*E_th_*) was approximately 3.5 V/μm. The *J_max_* was only 2.6 mA/cm^2^, with emission current fluctuation within 10% over 180 mins [12]. Another type of graphene used as the cathode material is directly deposited on the surface of a metal substrate using the chemical vapor deposition (CVD) method [14,15]. CVD graphene possesses excellent thermal and electrical conductivity as well as superior stability. However, this graphene usually lies flat on the substrate surface with little microscopic protrusions, resulting in a low field enhancement factor and, consequently, limited FE current.

To further enhance the emission performance of CVD graphene cathodes, researchers have utilized plasma-enhanced CVD (PECVD) to prepare vertically oriented graphene and studied its FE properties [16]. Zhang et al. grew vertically standing multilayer graphene films on a silicon substrate, with an *E_to_* of 1.8 V/µm and a current density of 7 mA/cm^2^ at 4.0 V/µm [17]. Jiang et al. prepared vertically oriented graphene arranged on a Cu substrate, also displaying a low Eto of 1 V/µm and a low Eth of 3 V/µm, with a high *β* (~11,000) [18]. It is obvious that compared to flat graphene, vertically oriented graphene demonstrates superior FE capabilities. However, the preparation process of this graphene is quite intricate and costly, which is not conducive to large-scale application.

In our previous studies, high-quality wrinkled multilayer graphene (MLG) was prepared using a cheap bubble chemical vapor deposition (B-CVD) method [19]. This MLG shares similar properties with CVD MLG, namely, high thermal and electrical conductivity and stability. Moreover, similar to vertically oriented graphene, this MLG also possesses abundant wrinkles and protruding structures, resulting in a large *β*, leading to good potentiality for FE cathode application. Therefore, in this study, we intend to utilize the MLG as the cathode material and systematically investigate its intrinsic emission properties and the influence of the cathode fabrication process on the FE performance.

## 2. Materials and Methods

### 2.1. Preparation of MLG Powder

MLG was prepared using the B-CVD method. The detailed preparation process is described in reference [19]. In brief, the B-CVD method employs molten Sn as a catalyst. During the growth process, methane gas is introduced into the molten Sn, forming methane bubbles. At a temperature of 1250 °C, methane undergoes high-temperature pyrolysis inside these bubbles, producing carbon atoms and hydrogen gas. The carbon atoms further assemble into high-quality MLG on the bubbles’ surface. As bubbles continue to form in the molten metal, MLG continues to grow. After 2 h of growth, the conversion efficiency of methane to MLG is 70 ± 10%. Therefore, this method enables the cost-effective production of high-quality MLG powder.

### 2.2. Preparation of MLG Cathode

Using the MLG powder, the cathodes were prepared through spin-coating and screen-printed methods, respectively.

#### 2.2.1. Cathode Preparation Using the Spin-Coating Method

MLG was added to N-methyl-2-pyrrolidone (NMP) at a concentration of 10 mg/mL and mixed with an equal mass of polyvinylpyrrolidone (PVP) as a surfactant to form a mixture. The mixture was magnetically stirred for 3 h and then sonicated for 30 mins to obtain uniform MLG dispersion. A silicon wafer was chosen as the substrate, with dimensions of 1 × 1 cm^2^ and a thickness of 500 μm. The silicon substrate was sequentially sonicated in acetone, ethanol, and deionized water for 10 min each to remove surface impurities, followed by drying in a vacuum oven at 80 °C for 1 h. The MLG dispersion was uniformly spin-coated onto the silicon substrate to obtain an MLG thin film using a spin coater with a maximum speed set at 4000 rpm. Finally, the sample was dried in a vacuum oven for 30 min, followed by annealing at 350 °C for 1 h in a muffle furnace to remove impurities and residual organic solvents to obtain the MLG cathode.

#### 2.2.2. Cathode Preparation Using the Screen-Printed Method

Ethyl cellulose and terpineol were mixed in a 1:13 ratio; the mixture was magnetically stirred for 4 h to obtain a transparent viscous organic adhesive. MLG powder (300 mg) was mixed with the organic adhesive (5.7 g) via magnetic stirring to obtain MLG paste. A 350-mesh polyester screen was used to print paste on a 1 × 1 cm^2^ silicon wafer. Subsequently, the sample was dried at 120 °C for 30 mins to enhance the adhesion between the MLG and the silicon wafer. Then, it was annealed at 200 °C for 10 min to remove organic impurities and the MLG cathode was obtained. (The reason for not choosing the same annealing temperature (350 °C) as that for the spin-coating cathode is because the organic adhesive contained in the MLG paste for screen printing would decompose at around 240 °C).

To further enhance the adhesion between MLG and the substrate, a silver paste buffer layer was screen printed on the substrate. In the experiment, DJ912 type conductive silver paste, composed of 65% conductive fillers, compounded resin, diluent, and additives, was used. After screen-printing, a sequential drying and annealing treatment was performed to remove organic impurities from the silver layer. The treatment process was as follows: the substrate with the silver layer was first dried at 120 °C in a vacuum oven for 40 min, followed by annealing it in a muffle furnace at different temperatures (200 °C, 350 °C, 400 °C). After cooling, another layer of MLG film was screen printed on the silver layer. The sample was then dried at 120 °C for 30 min and annealed at 200 °C for 10 min to obtain the MLG cathode. The cathodes with the silver layer annealed at 200 °C, 350 °C, and 400 °C were labeled as T-200, T-350, and T-400, respectively.

### 2.3. Field Emission Measurement Method

**Figure 1:**
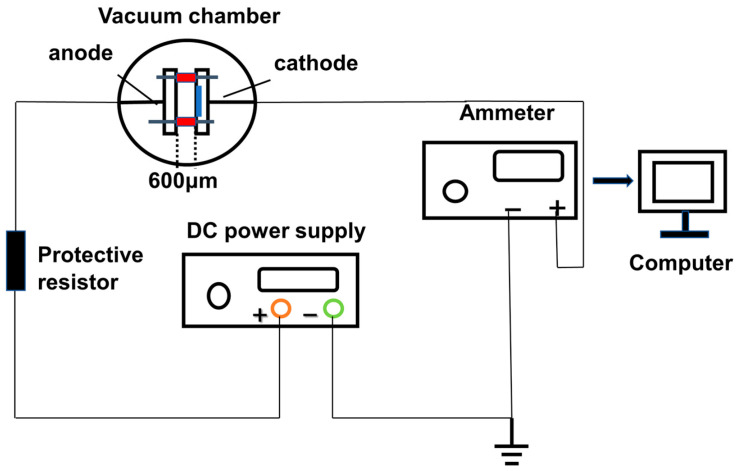
Circuit diagram for FE testing.

The FE performance of the cathode was evaluated in a diode with a cathode–anode gap of 600 μm in a vacuum environment of 8 × 10^−4^ Pa. The testing circuit is shown in Figure 1. The HPS1218 high-voltage DC power supply (Hangzhou Blue Instrument Co., Ltd., Hangzhou, China) with a voltage range of 0–6 kV and a maximum output power of 1200 W was used as the power source. The cathode emission current was detected and recorded using a Keithley 2400 digital source meter (Keithley Instruments, Cleveland, OR, USA). Before each FE measurement, the MLG cathode is initially held at a voltage of 1000 V for aging for 5 min. The turn-on field (*E_to_*) and threshold field (*E_th_*) of the cathode were defined as the electric field strengths required to achieve current densities of 10 μA/cm^2^ and 1 mA/cm^2^, respectively. The maximum current density (*J_max_*) is defined as the maximum current density that can be measured before breakdown occurs.

### 2.4. Materials Characterization Methods

The microstructure of the MLG powder and cathode was analyzed using a field emission scanning electron microscope (FE-SEM, FEI Quattro S (FEI Corporation, Eindhoven, The Netherlands)) at an acceleration voltage of 10 kV. Raman spectra of MLG were acquired using a WITec Alpha 300 R Raman spectrometer (WITec, Ulm, Germany), with a 532 nm laser source. The prepared MLG, after undergoing ethanol sonication, was deposited onto a porous copper grid for transmission electron microscopy (TEM) analysis. The FEI Tecnai G2 F20 S transmission electron microscope (FEI Corporation, Eindhoven, The Netherlands) with an accelerating voltage of 80 kV was used to produce transmission electron microscope images and selected area electron diffraction (SAED) patterns. The crystallinity of the prepared MLG was assessed through X-ray diffraction (XRD) analysis using a Bruker AXS D8 ADVANCE X-ray diffractometer (Bruker, Billerica, MA, USA) equipped with a Cu Kα (λ = 1.5418 Å) radiation source, operating at 40 kV and 40 mA and scanning at a rate of 2°/min with a step size of 0.02°. The elemental composition of MLG was analyzed using an energy-dispersive X-ray spectrometer (EDAX ELECT PLUS, Mahwah NJ, USA) operating at a voltage of 20 kV. The X-ray photoelectron spectra (XPS) of the MLG were obtained using a Thermo-Scientific Nexsa X-ray Photoelectron Spectrometer (Thermo-Scientific, Waltham, MA, USA) with the Al Kα X-ray radiation (hv = 1486.68 eV; spot size = 400 μm) source. The Fourier transform infrared (FTIR) spectra of MLG were obtained using the Thermo Fisher Nicolet Is10 infrared spectrometer (Thermo Fisher, Waltham, MA, USA). The spectra were collected in the wave number range from 4000 to 400 cm^−1^. Thermogravimetric analysis was performed under a nitrogen atmosphere and at a pressure of 1 atm using a thermogravimetric analyzer (Mettler Toledo TGA 2, Wetherill Park, NSW, Australia). The analysis covered a temperature range of 80–800 °C, with a temperature ramp rate of 20 °C/min.

## 3. Results and Discussion

### 3.1. MLG Characterization Results

**Figure 2:**
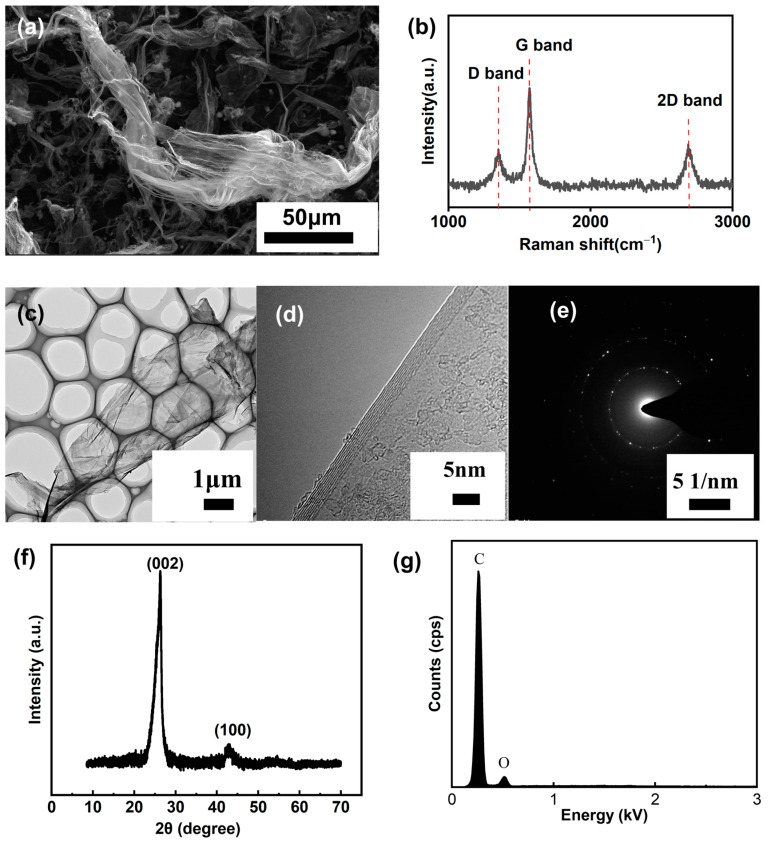
(**a**) SEM images of MLG powder; (**b**) Raman spectrum of MLG powder; (**c**) TEM and (**d**) HRTEM image of MLG; (**e**) SAED pattern of MLG; (**f**) XRD spectrum of MLG; (**g**) EDAX results of MLG.

**Figure 3:**
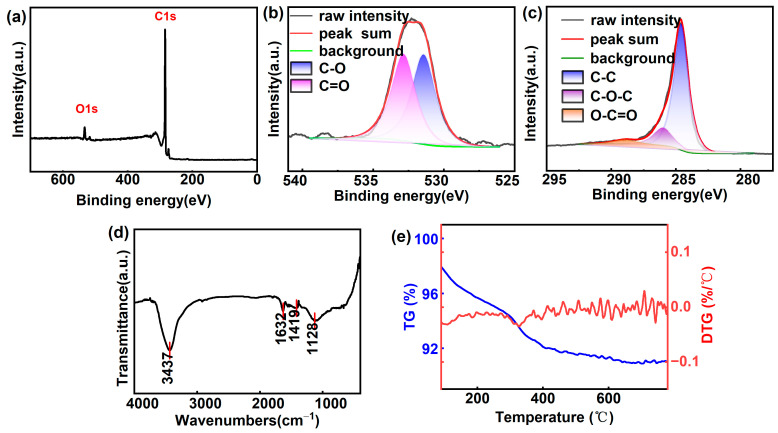
(**a**) XPS spectrum of MLG; (**b**) deconvolution of C1s spectrum of MLG; (**c**) deconvolution of O1s spectrum of MLG; (**d**) FTIR spectrum of MLG; (**e**) TG (blue) and DTG (red) plots of MLG.

The morphology of the MLG was characterized via SEM (Figure 2a). The surface of MLG exhibits a ribbon-like wrinkled structure with extensive protrusions, serving as effective emission sites for FE. The Raman spectrum of the MLG is presented in Figure 2b. The quality of MLG is evaluated by the ratio of the intensities of the D peak to the G peak (*ID/IG*), indicating the degree of defects or disorder [20]. The low *ID/IG* ratio (0.39) of MLG used in this study suggests excellent quality, i.e., fewer defects and therefore higher thermal and electrical conductivity. Figure 2c,d display the TEM and HRTEM results of the MLG, further confirming the wrinkled structure and pronounced edges of the MLG. The SAED results (Figure 2e) further reveal that MLG has a high crystallinity, indicating that the graphene is of good quality. Figure 2f shows an X-ray diffraction (XRD) pattern of the MLG sample, showing a sharp peak centered on 2θ = 26.3°, indicating excellent crystallinity. The EDAX result (Figure 2g) confirms the presence of oxygen, indicating partial oxidation of the MLG. Figure 3a present the XPS result for the MLG. The peak corresponding to C1s is observed at a banding energy of 284 eV, confirming the presence of sp^2^ hybridized carbon atoms [21]. Additionally, the peak at 532 eV, corresponding to O1s, further indicates the presence of oxygen in the carbon network structure [22]. The deconvoluted C1s and O1s spectra are shown in Figure 3b, c, both indicating the presence of C–O and C=O bonds on the MLG. The oxygen content is 5.47% and the C/O ratio is 17.282. Figure 3d depicts the FTIR spectrum of MLG, with the most prominent peak observed at 3437 cm^−1^, corresponding to the stretching vibration of O–H groups from water molecules and hydroxyl groups [23]. A peak around 1420 cm^−1^ corresponds to the deformation vibration mode of O–H bonds [23]. These peaks indicate the existence of hydroxyl groups and water molecules in the sample. The peak at 1128 cm^−1^ is the stretching vibration peak of C–O–C [24], suggesting the presence of oxygen-containing functional groups, which may originate from oxidation reactions occurring during sample preparation or purification processes. The peak observed at 1632 cm^−1^ corresponds to the C=C skeletal vibration of the graphene plane [25]. Figure 3e illustrates the thermogravimetric (TG) and derivative thermogravimetric (DTG) curves of the MLG sample. Within the range of 100–400 °C, a decrease in weight can be observed, which is primarily due to the removal of oxygen-containing functional groups [26].

### 3.2. Field Emission Performance

#### 3.2.1. Field Emission Performance of Spin-Coating Cathodes

**Figure 4:**
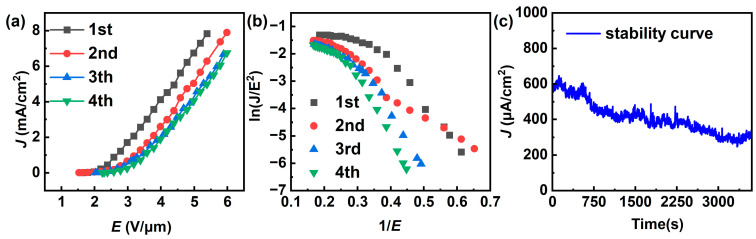
(**a**) The *J*–*E* and (**b**) F–N curves for the MLG cathode prepared via the spin-coating method. (**c**) Current stability test of the MLG cathode prepared via the spin-coating method.

The FE performance of the bare Si substrate was evaluated first, and no emission current could be detected at an applied field ranging from 0 to 6 V/μm, indicating that the substrate had no noticeable impact on the FE characteristics of MLG cathodes. With this conclusion, the MLG cathode prepared via the spin-coating method was measured. The *J* (field emission current density)–*E* (electric field strength) curves were collected for four voltage cycles until breakdown occurred. The *J–E* curves are shown in Figure 4a, and the *E_to_*, *E_th_*, and *J_max_* for four cycles are presented in Table 1. The *E_to_*/*E_th_* for the cathode are 1.63/2.68, 1.53/3.18, 2.03/3.38, and 2.23/3.58 V/μm, respectively, with a *J_max_* reaching 7.9 mA/cm^2^. The high current density indicates excellent intrinsic emission performance of the MLG material.

The FE characteristics of the cathode can be described by the F–N equation [1]:

(1) ln(JE)=ln(Aβ2φ)−Bφ32βE

where J is the cathode current density, *E* is the macroscopic electric field, A and B are constants, where $A=1.56\times 10^{-6}{\;\mathrm{AV}}^{-2}\mathrm{eV}$ and $B=6.83\times 10^{3}{\;\mathrm{VeV}}^{-\frac{3}{2}}\mu m^{-1}$, and *β* and *φ* represents the cathode’s field enhancement factor and work function, respectively. In our case, the work function of MLG is taken as 5 eV [27]. Typically, the F–N plot represents the relationship between ln(*J*/*E*) and 1/*E*. Assuming the slope of the F–N curve is denoted as *k*, the field enhancement factor can be expressed as β=Bφ32/k. Figure 4b shows the F–N curve of the cathode, indicating an approximately linear trend, signifying that the emitted current is due to the FE of MLG. By fitting the F–N curve, the calculated *β* for each of the four cycles was 7888, 8492, 5816, and 4568, respectively. It is observed that the FE performance deteriorates with increasing test cycles. Figure 4c illustrates the FE stability of the spin-coating MLG cathode at a constant voltage of 1470 V. The initial current density is approximately 600 μA/cm^2^, and after 1 h of testing, a decay of about 50% in current density is observed, further highlighting the poor stability of this cathode.

**Figure 5:**
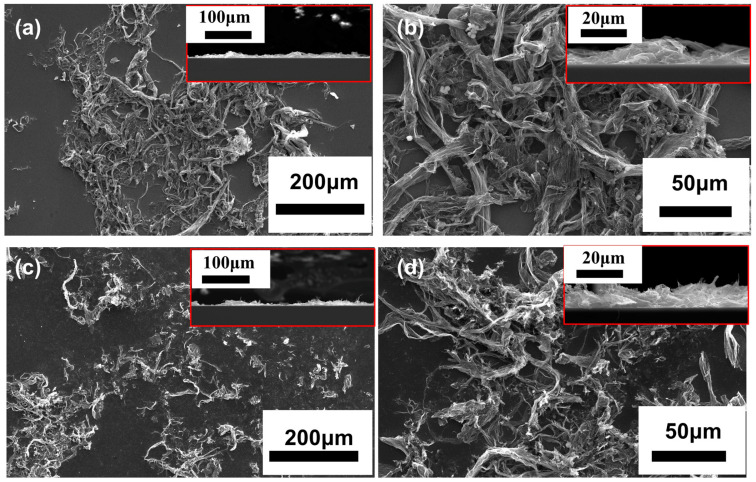
SEM images of s spin-coating MLG cathode: (**a**,**b**) before testing; (**c**,**d**) after testing. Insets show the corresponding cross-sectional morphologies.

To explore the reasons for the poor stability of these MLG cathodes, we conducted an analysis on the cathode micromorphology before and after the emission tests. Figure 5a,b depict the morphology of the cathode before the emission tests, and Figure 5c,d show the morphology of the cathode after four cycles. It can be observed that the spin-coating MLG maintains its wrinkled structure, ensuring enough effective field emission tips. Consequently, the *J_max_* of the cathode reaches 7.9 mA/cm^2^. The poor reproducibility of the cathode is due to (1) the MLG deposited by the spin-coating method having weak adhesion to the substrate, making the MLG film prone to detachment during field emission tests; and (2) the MLG dispersion on the surface of the cathode being inhomogeneous, resulting in localized excessive emission currents and overheating, accelerating surface damage and detachment of the MLG from the substrate. As shown in the inset in Figure 5d, even though the MLG tips becomes sharper after the four cycles, the reproducibility and stability remain poor due to partial detachment of, and damage to, the MLG.

In summary, the MLG cathode prepared via spin coating exhibits excellent intrinsic FE performance. However, the adhesion between the MLG film and the silicon substrate is weak and the uniformity of the MLG is poor.

#### 3.2.2. Field Emission Performance of Screen-Printed Cathodes

**Figure 6:**
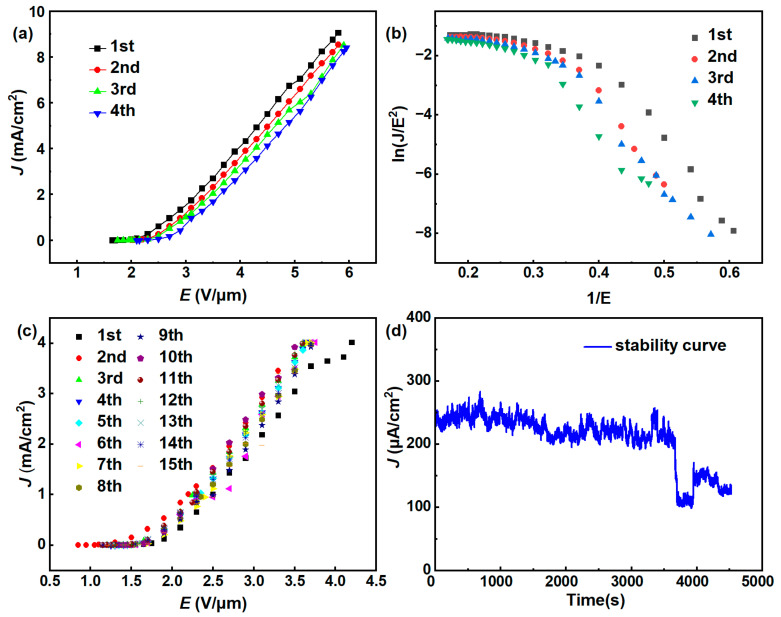
(**a**,**b**) *J*–*E* and F–N curves of screen-printed MLG cathode; (**c**) *J*–*E* curves from 15 FE cycles (with a limited *J* of 4 mA/cm^2^); (**d**) current stability test at 1330 V.

The FEs of the screen-printed cathodes were evaluated under the same conditions as illustrated in Section 3.2.1. The *J–E* and F–N curves are shown in Figure 6a, b. Table 2 presents the *E_to_*, *E_th_*, and *J_max_*. It can be observed that the cathode has better reproducibility. However, there is still noticeable performance degradation with increasing test cycles. The *E_to_* increased from 1.85 V/μm to 2.15 V/μm, and the *E_th_* increased from 2.7 V/μm to 3.1 V/μm. The *β* values for the four cycles were 5427, 5503, 4561, and 4825, respectively. The deterioration may be caused by excessive current density leading to gas ionization, resulting in breakdown and damage to the cathode [28]. To further confirm the reason for the deterioration, 15 voltage cycles were conducted with a limited *J* of 4 mA/cm^2^ where the breakdown never happened. It can be seen that after 15 cycles (Figure 6c), there was no significant change in the *J–E* curve, indicating that the deterioration was indeed caused by breakdown, and the stability of the cathode is satisfactory at a lower current density.

**Figure 7:**
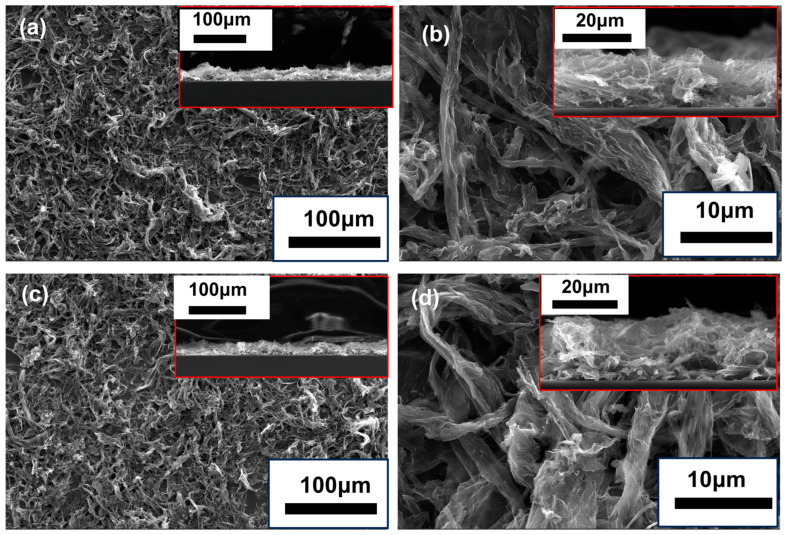
SEM images of the MLG cathode prepared via the screen-printed method: (**a**,**b**) before testing; (**c**,**d**) after testing. Insets are corresponding cross-sectional morphologies.

The microstructure of the cathode before and after the tests was also characterized. Figure 7a,b display the morphology of the cathode before testing, while Figure 7c,d show the morphology after 15 voltage cycles. It can be observed that after 15 cycles, no significant changes in the morphology can be observed, indicating passable stability.

Figure 6d illustrates the stability of the cathode at a DC voltage of 1330 V. The initial current density was approximately 250 µA/cm^2^. Within the first hour, the emission current density slightly decreased. Thereafter, a steep decline was observed. The stability of this cathode was better than that of the cathode prepared via spin coating. Nonetheless, degradation in emission performance still occurred. This could be attributed to the high contact resistance between the silicon substrate and the MLG [29,30], which generates additional Joule heating during field emission, accelerating gas desorption from the cathode, leading to ionization. Ion bombardment on the cathode during ionization can cause erosion at the emission sites, affecting the cathode’s lifetime [28].

#### 3.2.3. Field Emission Performance of Screen-Printed Cathodes with Silver Buffer Layer

To further enhance the stability of the cathode, a silver buffer layer was applied between the silicon substrate and the MLG film using the screen-printing method. This silver layer can not only strengthen the adhesion between MLG and the substrate but also reduce the contact resistance between them, facilitating electron transport and minimizing Joule heating [31].

**Figure 8:**
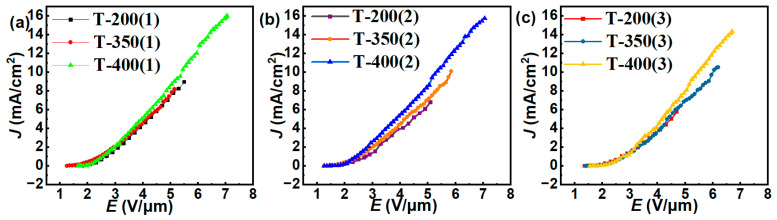
The comparative *J*–*E* curves of samples T-200, T-350, and T-400, respectively for (**a**) the first testing cycle; (**b**) the second testing cycle; (**c**) the third testing cycle.

We conducted three FE cycles on samples T-200, T-350, and T-400 with the silver buffer layer until breakdown occurred. The resulting *J–E* curves are depicted in Figure 8, and Table 3 presents the values of the *E_to_* and *E_th_* of different samples. The *J_max_* of T-400, T-350, and T-200 are 16.00 mA/cm^2^, 10.50 mA/cm^2^, and 8.96 mA/cm^2^, respectively. Figure 8 also indicates that there is little deviation in the three *J–E* curves for each cathode.

**Figure 9:**
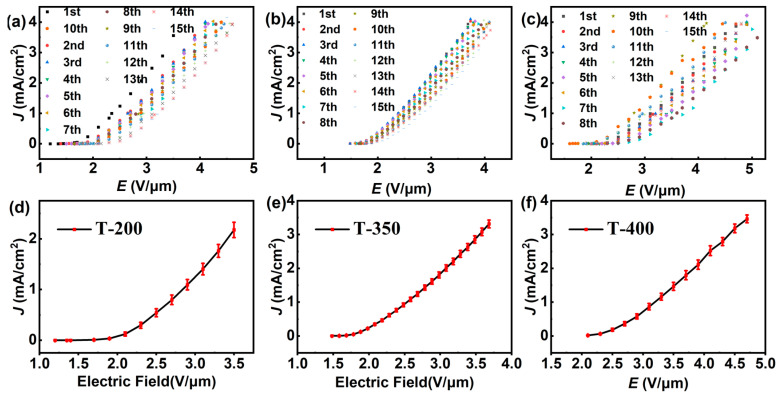
*J–E* curves of (**a**) T-200, (**b**) T-350, and (**c**) T-400, respectively (with a limited *J* of 4 mA/cm^2^). The statistical mean values and the standard deviation of the FE test cycles of the (**d**) T-200, (**e**) T-350, and (**f**) T-400 samples.

To further validate the reproducibility of the cathodes, each cathode was tested for 15 cycles with a limited *J* of 4 mA/cm^2^ (Figure 9a–c). Figure 9d–f depict the statistical mean values and standard deviation of the 15 cycles. By comparison, it can be found that T-350 exhibits the best repeatability and stability. The results show that the cathodes with the silver layer, especially for the T-350 sample, are more stable.

**Figure 10:**
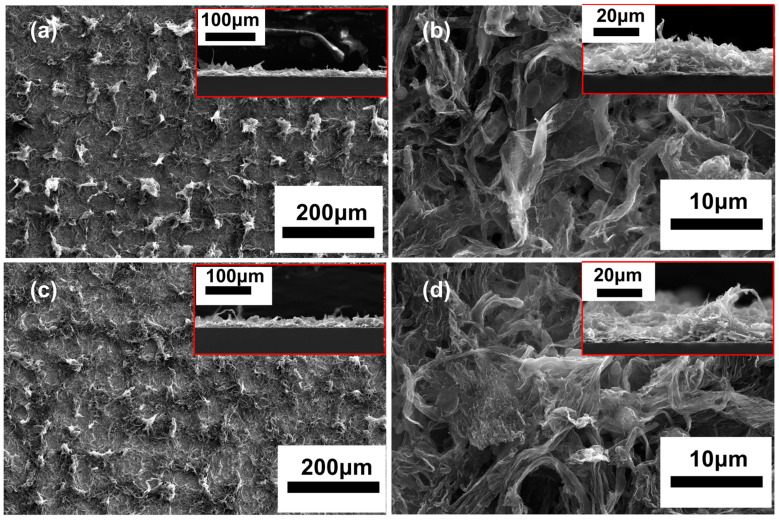
T-200 frontal SEM images: (**a**,**b**) before testing; (**c**,**d**) after testing. Inset: cross-sectional SEM images corresponding to T-200.

To elucidate the reason, the surface morphologies of these cathodes were also investigated. Figure 10a,b depict the morphologies before testing, while Figure 10c,d represent the morphologies after 15 cycles. In comparison to cathodes without the silver layer, these cathodes exhibit a pronounced grid-like distribution on the surface. The MLG protrudes on these grids, firmly anchored to the silver layer at the bottom. This results in a strong adhesion to the substrate, leading to enhanced stability and reduced contact resistance.

**Figure 11:**
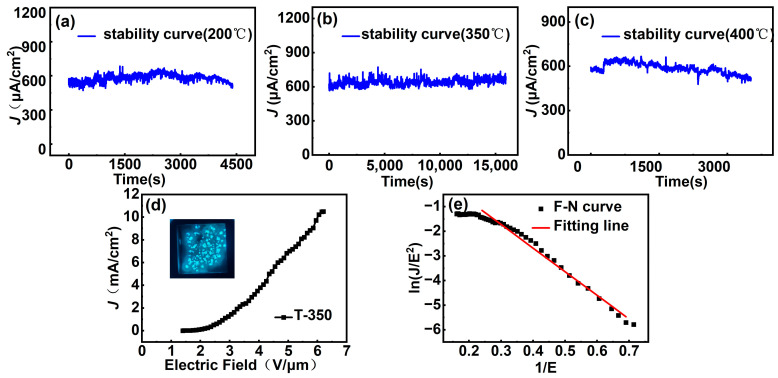
FE stability tests of (**a**) T-200, (**b**) T-350, and (**c**) T-400. (**d**) The *J–E* curve for the FE of T-350; the illustration is the luminescence pattern of the screen-printed MLG cathodes. (**e**) The F–N curve for the FE of T-350.

To further evaluate the influence of the annealing temperature of the silver buffer layer on the lifetime of cathodes, the field emission performances of T-200, T-350, and T-400 were tested under DC voltages. As shown in Figure 11a–c, T-200 and T-400 exhibit deterioration on the emission current, while T-350 maintains a relatively constant current in 4 h. Formula (2) is used to evaluate the fluctuation of current density, where “p” represents the fluctuation of current density, $I_{end}$ denotes the final current value, and $I_{ave}$ represents the average current value. When “p” is negative, it indicates a decay in the final emission current, while a positive value indicates an increased final emission current. The “p” values for T-200, T-350, and T-400 are −14.97%, +6.28%, and −13.12%, respectively. The FE current of both T-200 and T-400 decreased, while the current of T-350 has the smallest fluctuation and even slightly increases.

(2) $$p=\frac{I_{end}-I_{ave}}{I_{ave}}\times 100\%$$

The difference in the FE performance of the T-200, T-350, and T-400 samples may be caused by the different amounts of volatile organic adhesive left in the silver layer. With higher annealing temperature, less organic adhesive is left, leading to a decreased amount of gas molecules released from the silver layer during the FE process and thus a higher breakdown threshold, i.e., higher *J_max_*. However, less adhesive also results in weaker adhesion between the silver layer and the MLG, leading to increased contact resistance between the silver layer and the MLG film. This can cause significant Joule heating, damaging the cathode. Consequently, the stability and reproducibility of the T-400 sample are inferior to those of T-350. The T-350 sample exhibits the best overall performance, with a *J_max_* of 10.5 mA/cm^2^, an *E_to_* of 1.5 V/μm, an *E_th_* of 2.65 V/μm, a *β* of 8154 (Figure 11e), and an excellent emission uniformity, as is shown in the inset in Figure 11d.

Table 4 compares our sample with other works. It is evident that our sample demonstrates superior FE performance. This indicates that the B-CVD MLG holds good potentiality for industrial FE application.

## 4. Conclusions

In this study, high-quality wrinkled MLG was used to prepare the FE cathode. The influence of the preparation processes on the emission performance was investigated. The research results indicate that cathodes prepared using the screen-printed method exhibit better emission stability compared to samples prepared using the spin-coating method. Furthermore, a silver paste buffer layer between the substrate and the MLG can significantly improve the emission performance of the cathode. The optimized cathode has a low turn-on field of 1.5 V/μm and a low threshold field of 2.65 V/μm. The maximum current density of the cathode is 10.5 mA/cm^2^, with a current fluctuation of 6.28% within 4 h, demonstrating good stability. Additionally, the cathode also exhibits excellent emission uniformity. Our study demonstrates that the high-quality wrinkled MLG is a promising field emission cathode material for industrial applications.

## Figures and Tables

**Table 1:** Repetitive tests of MLG prepared via the spin-coating method.

Number of Cycles	*E_to_* (V/μm)	*E_th_* (V/μm)	*J_max_* (mA/cm^2^)
1	1.63	2.68	7.8
2	1.53	3.18	7.9
3	2.03	3.38	6.7
4	2.23	3.58	7.4

**Table 2:** Reproducibility testing of the MLG cathode prepared via the screen-printing method.

Number of Cycles	*E_to_* (V/μm)	*E_th_* (V/μm)	*J_max_* (mA/cm^2^)
1	1.85	2.7	9.057
2	2.05	2.9	8.543
3	2.05	3	8.516
4	2.15	3.1	8.409

**Table 3:** *E_to_* and *E_th_* of cathodes at different annealing temperatures.

Samples	Annealing Temperature for Silver Buffer	*E_to_* (V/μm)			*E_th_* (V/μm)		
		1st	2nd	3rd	1st	2nd	3rd
T-200	200 °C	1.65	1.5	1.45	2.65	2.75	2.7
T-350	350 °C	1.25	1.35	1.5	2.45	2.45	2.65
T-400	400 °C	1.72	1.47	1.7	2.55	2.45	2.75

**Table 4:** Summary of the FE characteristics of MLG cathodes in various studies in the literature.

Samples	*E_to_* (MV/m)	*E_th_* (MV/m)	*β*	*I_max_* (mA)	*J_max_* (mA/cm^2^)	Ref.
Screen printing	1.5 (1 μA/cm^2^)	3.5 (1 mA/cm^2^)	4539	2.6	2.6	[12]
Screen printing	1.56 (10 μA/cm^2^)	5.12 (10 mA/cm^2^)	3892	-	39.8	[32]
Electrophoretic deposition	2.3 (10 μA/cm^2^)	5.2 (10 mA/cm^2^)	3700	0.18	22.5	[33]
Microwave-plasma-enhanced CVD	2.4 (10 μA/cm^2^)	3.5 (1 mA/cm^2^)	4500	0.13	4.2	[34]
Microwave plasma CVD	1.3 (10 μA/cm^2^)	3 (1 mA/cm^2^)	11,000	0.83	1.3	[17]
RF sputtering	2.522 (10 μA/cm^2^)	3.401 (1 mA/cm^2^)	1781	-	4	[35]
Cold pressing (G-280,30st)	1.48 (100 μA/cm^2^)	1.9 (10 mA/cm^2^)	4820	4.12	515.46	[36]
Spin coating	4 (10^−2^ μA/cm^2^)	-	1200		1	[11]
This work	1.5 (10 μA/cm^2^)	2.65 (1 mA/cm^2^) 6 (10 mA/cm^2^)	8154	10.5	10.5	

## Data Availability

The data that support the findings of this study are available from the corresponding author upon reasonable request.
